# Domestic Correspondence

**Published:** 1889-03

**Authors:** 


### Domestic Correspondence.

To the Editor:

Recent events in the First District Society of New York point unmistakably to the need that exists for a revision of the present meagre rules of professional conduct, called the Code of Ethics. There are many recent evidences that as a class we have been awaking to a realization of our deficiencies in our practical relation, while claiming to be professional men.

The influence and power which trade has been gradually acquiring is now apparent to many who for long have been either blind or indifferent. The hindrances so long unnoticed are beginning to be felt, and "ethics in materials and methods" are beginning to be appreciated. In a more formal way the ethics of fraternal relations has been a subject for serious discussion in the above society. Charges were preferred by one member against another for violation of the "Code of Ethics" and unprofessional conduct, in a case in which a patient of the former was referred to the latter for special treatment of a "sore mouth." The offense consisted in the latter dentist going further than was intended or desired by the former, in making for the patient an artificial denture, after treating the mouth, without consulting with, or informing the dentist who had sent the patient to him. It was understood to have been shown before the committee of investigation, that the patient *desired* the dentist who treated the mouth to make the denture, and that she declared she would not in any case return to him who had referred her for treatment, and that this declaration, with the repeatedly expressed wish, seemed to be the inducement that led to the action complained of. It was also understood that the committee were prepared to report to the society in favor of the defendant, *not proven*, on the ground that the "Code of Ethics" had nothing in it bearing on this case or any similar one, which on reference proves to be true.

The defendant, however, through conviction of error, or unwillingness to trust the temper of the society, formally apologized to his accuser immediately before the meeting which was to have received and acted on the committee's report. So that when the meeting was convened, the announcement was made that such apology had been tendered and received, and the charges withdrawn. Clearer views of fraternal privileges and duties will undoubtedly be formed as a result of the incident, and in this way good has been accomplished; but the inadequate provisions of the

"Code," "adopted from the Code of the American Dental Association," is made so apparent that it may well be hoped that it will lead to a general revision of the code of dental societies, or else to their entire obliteration; so that the accepted rules of professional conduct may be commensurate to ordinary requirements, or that the general moral sense may be left to judges of ethical standards, without being hampered by formal standards of law, which are so insufficient. * * *

---

To the EDITOR:

When Dr. C. E. Francis introduced "Incidents of Office Practice" as a heading in a society's order of business, he did a good thing for us all. May I not give you an incident? A skillful plate workman last summer asked me why I made my clasps for partial plates so narrow, and why I attached them to the plate by a narrow standard.

He said that a narrow clasp would inevitably wear the tooth to which it was attached, and that I would soon find a groove under the clasp just the size of the clasp.

I replied that the groove was generally found whatever the width of the clasp, but that it was never the result of wear, inasmuch as it would be found under a soft rubber clasp quite as surely as under a gold one.

"Yes," he said, "that's so; but what makes the groove?"

I replied, "the chemical action of fermenting food lodged between the clasp and the tooth, and the bigger the clasp and the broader its attachment to the plate, the larger the amount of food that continuously lodges there."

A patient, yesterday, returned to me after many years of absence. She had a plate in her mouth that I made sixteen and one-half years ago, clasping the bicuspid teeth.

One of those teeth I extracted, as it was too loose to remain longer; the clasp was still around it after all those years.

I send you clasp and tooth that you may see their conditions.

My own description of the case would be, that being so nearly self-cleansing, the clasp had done practically no harm to the tooth; for while a little wear is visible on one side, I think that damage has been counter-balanced by the rubbing which has tended to free the tooth from foreign deposits.

If clasps are put just above the tuberosity of the tooth where the enamel is thick (not up by the neck where it is thin), and are soldered by a narrow standard, leaving as much elasticity as possible, there is very little evil effect from a narrow clasp in a moderately cleanly mouth.

New York City. E. A. Bogue.

[Tooth and clasp were duly received and showed, as described, only very slight abrasion; so little, in fact, as to be quite remarkable after so long wear.—Ed.]

---

To the Editor:

Rubber dam clamps are among the most valuable instruments that a dentist uses; at the same time they are among them most dangerous. Any one who has used them for a long series of years has had cases where decay came as the result of the injury inflicted on the enamel by the hard and sharp edges of the steel jaws of the clamp. I have seen many such results in delicate teeth, and have sought for some means to overcome this. In my own practice I employ clamps with adjustable jaws of soft metal or other substances like hard rubber and celluloid. These jaws are easily replaced if injured, and enamel would require to be very soft, indeed, to receive injury from such clamps. I have never seen a case that showed subsequent decay from the use of my clamps. I describe them here in order to prevent, if possible, some unscrupulous person from patenting the invention, which consists briefly in making clamps with removable and adjustable jaws; said jaws, instead of being made as at present of hard steel, are constructed of soft metals like tin or rubber or celluloid, or other suitable material; the object being to provide jaws too soft to injure the enamel of the tooth. These little removable jaws can be molded in quantity, and are therefore cheap, and at the same time they are readily bent to fit any special form of the tooth to which they are to be applied; this is a strong point in their favor. Besides this advantage they require but a few clamps to fit a whole set of teeth, for the jaws being removable suitable ones can be selected from a great number and simply placed in the clamps for use.

Boston, Jan. 18, 1889. William Herbert Rollins.

---

A correspondent of the *New York World* (Nov. 4th) advocates the organization of a S. P. A. T.*

Judging from some signatures recently noted, we suggest that he endeavor to recruit his ranks from among the members of the dental profession, to the manifest advantage of the latter. A. B———c, A.M., M.D., D.D.S., F.R.S., M.S.P.A.T., for instance!

---

* Society for the Prevention of the Accumulation of Titles.

